# Association Between rs12037447, rs146732504, rs151078858, rs55723436, and rs6094136 Polymorphisms and Kawasaki Disease in the Population of Polish Children

**DOI:** 10.3389/fped.2021.624798

**Published:** 2021-02-22

**Authors:** Piotr Buda, Maciej Chyb, Anna Smorczewska-Kiljan, Anna Wieteska-Klimczak, Agata Paczesna, Monika Kowalczyk-Domagała, Magdalena Okarska-Napierała, Marta Sobalska-Kwapis, Łukasz Grochowalski, Marcin Słomka, Aneta Sitek, Janusz Ksia̧żyk, Dominik Strapagiel

**Affiliations:** ^1^Department of Pediatrics, Nutrition, and Metabolic Diseases, Children's Memorial Health Institute, Warsaw, Poland; ^2^Biobank Lab, Department of Molecular Biophysics, Faculty of Biology and Environmental Protection, University of Lodz, Łódź, Poland; ^3^Department of Cardiology, The Children's Memorial Health Institute, Warsaw, Poland; ^4^Department of Pediatrics With Clinical Decisions Unit, Medical University of Warsaw, Warsaw, Poland; ^5^BBMRI.pl Consortium, Wrocław, Poland; ^6^Department of Anthropology, Faculty of Biology and Environmental Protection, University of Lodz, Łódź, Poland

**Keywords:** Kawasaki disease, genome-wide association study, susceptibility gene, KIF25, PTPRJ, SPECC1L, RNP2

## Abstract

**Background:** Kawasaki disease (KD) is an acute self-limited febrile vasculitis that mainly affects young children. Coronary artery involvement is the most serious complication in children with KD. It is currently the leading cause of acquired cardiac disease in children from developed countries. Literature data indicate a significant role of genetic susceptibility to KD.

**Objective:** The aim of this study was to perform the first Genome-Wide Association Study (GWAS) in a population of Polish children with KD and identify susceptible genes involved in the pathogenesis of KD.

**Materials and Methods:** The blood samples of Kawasaki disease patients (*n* = 119) were collected between 2016 and 2020, isolated and stored at the Department of Pediatrics, Nutrition and Metabolic Diseases, Children's Memorial Health Institute in Warsaw. The control group was based on Polish donors (*n* = 6,071) registered as the POPULOUS collection at the Biobank Lab of The Department of Molecular Biophysics in University of Lodz. DNA samples were genotyped for 558,231 Single Nucleotide Polymorphisms (SNPs) using the 24 × 1 Infinium HTS Human Core Exome microarrays according to the protocol provided by the manufacturer. In order to discover and verify genetic risk-factors for KD, association analysis was carried out using PLINK 1.9.

**Results:** Of all 164,395 variants, 5 were shown to occur statistically (p_adjusted_ < 0.05) more frequent in Kawasaki disease patients than in controls. Those are: rs12037447 in non-coding sequence (p_adjusted_ = 8.329 × 10^−4^, OR = 8.697, 95% CI; 3.629–20.84) and rs146732504 in KIF25 (p_adjusted_ = 0.007354, OR = 11.42, 95% CI; 3.79–34.43), rs151078858 in PTPRJ (p_adjusted_ = 0.04513, OR = 8.116, 95% CI; 3.134–21.01), rs55723436 in SPECC1L (p_adjusted_ = 0.04596, OR = 5.596, 95% CI; 2.669–11.74), rs6094136 in RPN2 (p_adjusted_ = 0.04755, OR = 10.08, 95% CI; 3.385–30.01) genes.

**Conclusion:** Polymorphisms of genes KIF25, PTRPJ, SPECC1L, RNP2 may be linked with the incidence of Kawasaki disease in Polish children.

## Introduction

Kawasaki disease (KD) is an acute self-limited febrile vasculitis that mainly affects young children. It is currently the leading cause of acquired cardiac disease in children from developed countries, associated with an increased risk of coronary heart disease (1). Although KD was first described in 1967, the etiology of the disease remains unknown (2). It is believed that there is a genetic susceptibility to incorrect activation of the immune system, and oligoclonal immune response to bacterial, viral or other unidentified environmental factors, which results in damage to vascular endothelial cells and necrotizing vasculitis (3, 4). The disease is most common in young children, with most cases between 6 months and 5 years old, with predominance in males. The clinical manifestations of KD include high fever, polymorphic rash, swelling and redness of the hands and feet, changes of the lips and oral mucous membranes, cervical lymphadenopathy, and aseptic conjunctivitis. Since the inflammatory process affects all vessels, the clinical manifestation can also involve many other systemic symptoms. The atypical form of KD is increasingly recognized and defined according to the American Society of Cardiology (American Heart Association, AHA) (5). The only clinical symptom found may be fever and abnormalities in additional tests, which can cause diagnostic errors. In infants the diagnosis is particularly challenging and often late, and the disease is more frequently resistant to treatment (5, 6).

Majority of vascular compromise occurs in the coronary arteries. In about 20% of patients, vasculitis leads to coronary artery lesions (dilatation, aneurysm) that are the principal cause of acquired heart disease of children. To-date's literature data indicate a significant role of genetic susceptibility to KD (3, 4). Many genes and chromosomal regions have been identified through genome-wide association studies to have an association with KD. Polymorphisms in ITPKC, ORAI, STIM, CD40, BLK, FCGR2A, and CASP3 were among the most commonly identified as the susceptibility genes for KD (7–10). However, many other new risk loci have been recently found (NMNAT2, HCP5, ZFHX3, NAALADL2, NEBL, TUBA3C) that indicate that KD could be regarded as a multifactorial and polygenic (complex) disorder (11–14). We performed the first genetic studies in a population of Polish children with KD and identified susceptibility genes involved in the pathogenesis of KD that may provide new insights into diagnosis and treatment of this condition.

## Materials and Methods

### Control Group

The control group is based on polish donors recruited within the research project TESTOPLEK between 2010 and 2012 and is registered as the POPULOUS collection at the Biobank Lab of The Department of Molecular Biophysics, University of Lodz, Poland, which is currently registered in Directory (v. 4.0) of BBMRI-ERIC consortium under bbmri-eric:ID:PL_BLUL:collection:POPULOUS_BLUL registration number (15, 16) (https://directory.bbmri-eric.eu/). Samples were obtained from individuals without chronic or active diseases, based on a statement in the survey, therefore it can be marked as a control group. The control group can be considered homogenous within the same pattern as other populations within European countries, based on carried out mitochondrial DNA and Y chromosome variability studies (17, 18). It consists of 3 109 females and 2 962 males of which age ranged from 20 to 77 years old. This project had the approval from The University of Lodz's Review Board (32/KBBN-UL/I/2018), and all procedures were performed in accordance with the Declaration of Helsinki. A total of 6,071 participants were used as control group in the study. Samples of saliva were collected into Oragene OG-500 DNA collection/storage tubes (DNA Genotek, Kanata, Canada) from each individual. Genomic DNA from saliva samples was manually isolated from 500 μL using the manufacturer's instructions (PrepitL2P, PD-PR-052, DNA Genotek, Kanata, ON, Canada).

### Case Group

The blood samples of 119 patients, 73 males and 46 females diagnosed with Kawasaki disease were collected between 2016 and 2020, isolated and stored at Department of Pediatrics, Nutrition and Metabolic Diseases, Children's Memorial Health Institute, Warsaw, Poland. Patients' ages ranged from 3 months to 14 years old. All children came from Poland (Mazowieckie Voivodeship), there were no ethnic differences (homogeneous Caucasian population). The diagnosis of Kawasaki disease was established according to the American Heart Association (AHA) criteria (5). Samples can be differentiated into additional phenotypes groups (Table 1). Parents of all patients gave written consent for the participation of their children in the study “Searching for molecular markers related to Kawasaki disease.” This project has the approval from the Bioethics Committee at the Children's Memorial Health Institute Review Board. Genomic DNA was isolated from blood samples mostly using MagCore pipetting station (RBC Bioscience) with the MagCore Genomic DNA Whole Blood Kit (TK Biotech). Some of the samples were isolated manually using Genomic Midi AX kit (A&A Biotechnology).

**Table 1:** Summary of the case group phenotypes.

**Kawasaki disease (typical)**	**Kawasaki disease (atypical)**
95 pts	24 pts
**Negative effect of IVIG**	**Positive effect of IVIG**
32 pts	87 pts
**CAL**	**No CAL**
43 pts	76 pts

*IVIG, intravenous immunoglobulins; CAL, coronary artery lesions*.

### Quality Control

In order to be qualified for microarray genotyping, samples had to pass quality control. Samples with DNA concentration equal or >50 ng/μl were qualified for microarray genotyping. DNA concentration was measured using broad range Quant-iT™ dsDNA Broad Range Assay Kit (Invitrogen™, Carlsbad, CA, USA). All DNA samples passed quality control in PCR reaction for sex determination (19).

### Microarrays Analysis

DNA samples were genotyped using the 24 × 1 Infinium HTS Human Core Exome (Illumina Inc., San Diego, CA, USA) microarrays according to the protocol provided by the manufacturer.

### Pre-processing

Raw fluorescence intensities were loaded into GenomeStudio 2.0 with Genotyping Module (Illumina, Inc.) in order to do Quality Control. Six thousand one hundred ninety (6,071 controls, 119 cases) samples with Call Rate above 0.95 were included into further analysis. Variants with incorrect clustering, Multi-allelic SNPs, and polymorphisms located at X and Y chromosomes were excluded from the analysis. 323,583 remaining SNPs where further processed in PLINK 1.9 software (20). One hundred fifty-six thousand three hundred forty-one variants were removed due to minor allele threshold and an additional 2,847 variants were removed due to Hardy–Weinberg exact-test (21). Therefore, statistical analysis was carried out on 164,395 variants. StrandScript was then used to ensure forward strand orientation (22).

### Statistical Analysis

Statistical analysis was based on the chi-square statistic with odds ratio and 95% confidence interval for 2 × 2 contingency tables and was carried out in PLINK 1.9 (20, 23). Bonferroni correction was used to counteract the problem of multiple comparisons. Manhattan plot was generated using the Haploview (24).

### Prediction of Single Nucleotide Polymorphism Effect

To estimate the effect of polymorphism on protein function, *in silico* online tools such as PredictSNP^2^ (25) and SIFT (Sorting Intolerant From Tolerant) was used (26). PredictSNP^2^ allowed us to use five different prediction tools and compare the results. Prediction is based on tools for scoring the deleteriousness of single nucleotide variants such as: CADD- (Combined Annotation Dependent Depletion) based on a support-vector machine (SVM) classifier; DANN (deleterious Annotation of Genetic Variants using Neural Networks) is based on a deep neutral network classifier; FATHMM (Functional Analysis through Hidden Markov Models) is based on an SVM classifier; FunSeq2 based on a weighted scoring system that combines genetic, epigenetic, and gene expression information; GWAVA (Genome-Wide Annotation of Variants) is based on a random forest classifier. PredictSNP^2^ score is based on the tools described above (25). To assess the significance of a variant placed in non-coding sequence as a candidate associated with Kawasaki disease, the HaploReg v4.1 tool was used (27).

## Results

### Comparison of All Allele Frequencies in Kawasaki Disease Patients (*n* = 119) and Controls (*n* = 6,071)

**Figure 1:**
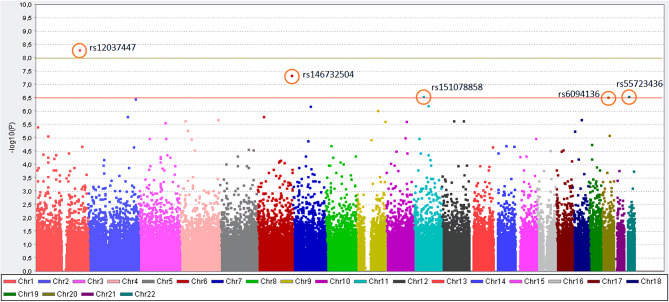
Manhattan plot for the genome-wide association study of patients with KD. The y-axis shows the- log10 of the unadjusted *p*-values (without Bonferroni correction). On the x-axis, each color represents different chromosomes, and the dots represent SNPs. The red line indicates the association threshold (10^−6.5^), which represents the corrected *p*-value = 0.05, while the green line indicates GWAS significant threshold (10^−8^).

Of all 164,395 variants, 5 were shown to occur statistically (p_adjusted_ < 0.05) more frequent in Kawasaki disease patients than in controls. Those are: rs12037447 in non-coding sequence (p_adjusted_ = 8.329 × 10^−4^, OR = 8.697, 95% CI; 3.629–20.84) and rs146732504 in KIF25 (p_adjusted_ = 0.007354, OR = 11.42, 95% CI; 3.79–34.43), rs151078858 in PTPRJ (p_adjusted_ = 0.04513, OR = 8.116, 95% CI; 3.134–21.01), rs55723436 in SPECC1L (p_adjusted_ = 0.04596, OR = 5.596, 95% CI; 2.669–11.74), rs6094136 in RPN2 (p_adjusted_ = 0.04755, OR = 10.08, 95% CI; 3.385–30.01) genes (Table 2 and Figure 1).

**Table 2:** Comparison of the frequency of SNPs in patients with KD disease (*n* = 119) and control groups (*n* = 6,071).

**CHR**	**SNP**	**POSITION**	**A1**	**F_A**	**F_U**	**A2**	**CHISQ**	***P***	**OR**	**SE**	**L95**	**U95**	**BONF**	**FDR_BH**	**Gene**	**Description**
1	rs12037447	208605593	G	0.02521	0.002965	A	34.16	5.066 × 10^−9^	8.697	0.4459	3.629	20.84	**8.329 × 10**^**−4**^	8.329 × 10^−4^	NCS	
6	rs146732504	168434704	A	0.01681	0.001494	C	29.93	4.474 × 10^−8^	11.42	0.5629	3.79	34.43	**0.007354**	0.003677	KIF25	Missense variant
11	rs151078858	48177395	T	0.02101	0.002637	C	26.42	2.745 × 10^−7^	8.116	0.4854	3.134	21.01	**0.04513**	0.009211	PTPRJ	Missense variant
22	rs55723436	24718408	A	0.03361	0.006177	G	26.39	2.796 × 10^−7^	5.596	0.3778	2.669	11.74	**0.04596**	0.009211	SPECC1L	Missense variant
20	rs6094136	35854009	G	0.01681	0.001693	A	26.32	2.892 × 10^−7^	10.08	0.5567	3.385	30.01	**0.04755**	0.009211	RPN2	Intron variant
2	rs139662037	227731962	C	0.01261	9.883 x10^−4^	T	26.03	3.362 × 10^−7^	12.9	0.6488	3.618	46.03	0.05527	0.009211	RHBDD1	Missense variant
11	rs7124405	70891161	T	0.5294	0.3713	C	24.9	6.027 × 10^−7^	1.905	0.1312	1.473	2.463	0.09908	0.01345	SHANK2	Intron variant
7	rs2662865	85463717	G	0.07203	0.02245	A	24.74	6.545 × 10^−7^	3.38	0.2599	2.031	5.625	0.1076	0.01345	NCS	
9	rs202207863	107289385	T	0.01261	0.001071	C	24.06	9.338 × 10^−7^	11.91	0.6439	3.372	42.07	0.1535	0.01706	OR13C4	Missense variant
6	rs9267431	31478951	A	0.2227	0.1194	G	23.08	1.552 × 10^−6^	2.113	0.1591	1.547	2.886	0.2551	0.02207	*Near* MICB	Downstream variant
2	rs12477499	189833034	G	0.02101	0.002965	A	23.02	1.605 × 10^−6^	7.215	0.4818	2.806	18.55	0.2638	0.02207	LOC105373791	Intron variant
4	rs148434007	185012401	T	0.01271	0.001153	C	22.55	2.05 × 10^−6^	11.15	0.6396	3.183	39.07	0.3371	0.02207	ENPP6	Missense variant
18	rs201067154	43535154	A	0.01681	0.001961	G	22.52	2.082 × 10^−6^	8.699	0.5495	2.963	25.54	0.3422	0.02207	EPG5	Missense variant
4	rs1013532	27157313	A	0.08824	0.03246	G	22.41	2.206 × 10^−6^	2.885	0.2342	1.823	4.565	0.3627	0.02207	NCS	
12	rs117650853	107360943	A	0.01261	0.001153	G	22.32	2.303 × 10^−6^	11.06	0.6396	3.157	38.74	0.3785	0.02207	TMEM263	Missense variant
12	rs201236531	60169242	A	0.01261	0.001153	G	22.32	2.308 × 10^−6^	11.06	0.6396	3.157	38.73	0.3794	0.02207	SLC16A7	Missense variant

*CHR, chromosome; POSITION, base pair based on the human reference genome GRCh37; A1, minor allele; A2, major allele; F_A, frequency of A1 allele in patients; F_U, frequency of A1 allele in controls; CHISQ, basic allelic test chi-square (1df); P, p-value for CHISQ; OR, odds ratio; SE, standard error; L95 and U95, 95% confidence interval for odds ratio, lower bound and upper bound, respectively; BONF, Bonferroni single-step adjusted p-values; FDR_BH, Benjamini and Hochberg step-up false discovery rate; NCS, non-coding sequence. Bold values are the variants discussed in the results section that were below p < 0.05*.

Other variants that did not pass, but were the closest to the established p_adjusted−_value <0.05 were: rs139662037 in RHBDD1 (p_adjusted_ = 0.05527, OR = 12.9, 95% CI; 3.618–46.03), rs7124405 in SHANK2 (p_adjusted_ = 0.09908, OR = 1.905, 95% CI; 1.473–2.463), rs202207863 in OR13C4 (p_adjusted_ = 0.1535, OR = 11.91, 95% CI; 3.372–42.07) genes and rs2662865 in non-coding sequence (p_adjusted_ = 0.1076, OR = 3.38, 95% CI; 2.031–5.625) (Table 2). Information about all variants, frequencies, and statistics in analysis is presented in Supplementary Table 1.

### Prediction of SNP Effect

The effect of five SNPs that showed to be the most statistically associated with the Kawasaki disease in this study was evaluated, this includes: rs12037447 in non-coding sequence and rs146732504 in KIF25, rs151078858 in PTPRJ, rs55723436 in SPECC1L, rs6094136 in RPN2 genes (Table 3). Rs55723436 is a missense variant G>A in the SPECC1L gene and was predicted as deleterious by all five used tools. Additionally, this result was confirmed by SIFT predictions in one of three possible outcomes of nucleotide change (Supplementary Table 2). A similar situation was observed in the case of rs151078858, a missense variant C>T in the PTPRJ gene, which was predicted as deleterious by four out of five tools use by PredictSNP^2^, and as unfavorable change by SIFT. On the other hand, rs146732504, a missense variant C>A in the KIF25 gene, and rs6094136 an intron variant A>G in the RPN2 gene, were classified as deleterious only by one of the tools CADD and GAWVA, respectively. However, rs146732504 was marked as deleterious by two out of three SIFT possible predictions, while rs6094136 was not found by this tool. Rs12037447is the only variant that was evaluated as neutral by all PredictSNP^2^ prediction tools and this is due to the location of this polymorphism. Therefore, to assess its effect, the HaploReg v4.1 tool was used. This SNP is placed in a non-coding sequence, but it appears to have regulatory functions. The results showed that this variant influences 16 regulatory motifs, potentially affecting various functions throughout the genome (Supplementary Table 3).

**Table 3:** Evaluation of SNP effect.

**Variant:**	**SNP outcome prediction**						
rs12037447 A>G	Tool:	PredictSNP^2^	CADD	DANN	FATHMM	FunSeq2	GWAVA
	Prediction:	neutral	neutral	neutral	neutral	neutral	neutral
	Score:	−1.0000	3.5990	0.5589	0.2198	0.4966	0.3700
	Exp. accuracy:	0.88	0.81	0.81	0.75	0.62	0.59
rs146732504 C>A	Tool:	PredictSNP^2^	CADD	DANN	FATHMM	FunSeq2	GWAVA
	Prediction:	neutral	**deleterious**	neutral	neutral	neutral	neutral
	Score:	−1.0000	25.1000	0.9101	0.8692	2.0000	0.2100
	Exp.accuracy:.	0.89	0.67	0.84	0.63	0.62	0.54
rs151078858 C>T	Tool:	PredictSNP^2^	CADD	DANN	FATHMM	FunSeq2	GWAVA
	Prediction:	neutral	**deleterious**	**deleterious**	neutral	**deleterious**	**deleterious**
	Score:	−0.0816	24.5000	0.9982	0.2557	3.0000	0.6500
	Exp.accuracy:.	0.65	0.63	0.70	0.84	0.61	0.51
rs55723436 G>A	Tool:	PredictSNP^2^	CADD	DANN	FATHMM	FunSeq2	GWAVA
	Prediction:	**deleterious**	**deleterious**	**deleterious**	**deleterious**	**deleterious**	**deleterious**
	Score:	1.0000	34.0000	0.9996	0.9698	3.0000	0.5900
	Exp.accuracy:	0.87	0.84	0.77	0.69	0.61	0.51
rs6094136 A>G	Tool:	PredictSNP^2^	CADD	DANN	FATHMM	FunSeq2	GWAVA
	Prediction:	neutral	neutral	neutral	neutral	neutral	**deleterious**
	Score:	−1.0000	2.0690	0.6916	0.1220	0.3459	0.5100
	Exp.accuracy:	0.88	0.83	0.62	0.85	0.81	0.65

*Prediction based on PredictSNP^2^. Bold values indicate Highlighting all deleterious results*.

## Discussion

We found that five single nucleotide polymorphisms were more commonly found in Polish children with Kawasaki disease than in adults: rs12037447 (non-coding region), rs146732504 (KIF25), rs151078858 (PTRPJ), rs55723436 (SPECC1L), rs6094136 (RNP2). Based on the currently available literature, none of them were previously linked to KD. We discuss below the function of proteins encoded by these genes, susceptibility to a variety of disorders and its potential role in the context of KD.

### KIF25

The protein encoded by gene KIF25 (also known as KNSL3) is a member of the kinesin-like protein family involved in the trafficking of vesicles, organelles, and proteins through the cytoskeleton in a microtubule- and ATP-dependent manner (28). This protein is a negative regulator of centrosome separation required to prevent premature centrosome separation during interphase. Intracellular transport is crucial for morphogenesis and functioning of the cell. Kinesins are proteins mainly expressed in neurons, immune cells, and oligodendrocytes. Dysregulation of kinesins has been postulated to aggravate multiple sclerosis disease: lower methylated levels for KIF25 have been measured in these patients in the hippocampi and CD4+ T cells (29–32). In the context of Kawasaki disease, its relation to KIF25 was not previously described. Only Zhang et al. found that specific exosomal miRNA (miR- 671-5p), downregulated in children with KD, is affecting the expression of kinesin family member 1B (33). Another kinesin—Kinesin Superfamily Motor Protein 4 (KIF4)—was found in the muscles of patients with idiopathic inflammatory myopathies and also in activated peripheral blood lymphocytes *in vitro* (34). Authors concluded that KIF4 is likely to be involved in the cytoskeleton modifications associated with T-cell activation, but further studies are required to elucidate the role of kinesin in inflammatory processes.

The particular function of this gene product has not yet been determined.

### PTRPJ (CD148)

Protein Tyrosine Phosphatase Receptor Type J is a protein encoded by gene PTRPJ. It is a member of the protein tyrosine phosphatase (PTP) family, signaling molecules that regulate cell growth, differentiation, mitotic cycle, and oncogenic transformation (35). PTRPJ is found in all hematopoietic lineages. This protein negatively regulates T cell receptor signaling possibly through interfering with the phosphorylation of Phospholipase C Gamma 1 and Linker for Activation of T Cells. PTPRJ/CD148 is a tyrosine phosphatase that has tumor suppressor-like activity due to its reduced expression in some malignant tumors, its regulation by cell density. PTPRJ polymorphisms were found to influence susceptibility to a variety of human cancers, e.g., thyroid, rectal colon cancers, meningiomas (36–39). Marconi et al. showed that biallelic-null mutations in PTPRJ gene impairs spreading on collagen, fibrinogen, and fibronectin; GPVI-mediated aggregation and secretion; and stromal cell-derived factor (SDF)-driven migration on fibronectin, all of which may contribute to thrombocytopenia (40). PTRPJ is also implicated in inflammatory disorders. The activation or enhancement of CD148 expression could lead to modulation of the inflammatory response. CD148 expression is upregulated in chronic inflammatory diseases, such as Crohn's disease and Cogan's syndrome (41, 42). Cogan's syndrome is a rare autoimmune vasculitis, characterized by sensorineural hearing loss, keratitis, and vasculitis. PTRPJ is expressed on the sensory epithelia of the inner ear and on endothelial cells. Antibodies to PTRPJ were identified in patients with Cogan's syndrome. Dave R et al. showed that macrophage-enriched tissues exhibit the highest expression of PTPRJ (43). CD148 is upregulated in response to various toll-like receptor ligands and downregulated by the colony-stimulating factor 1 (CSF1, macrophage colony-stimulating factor, M-CSF). There is an essential role for CSF1 and granulocyte/macrophage colony-stimulating factor (GM-CSF) in the pathogenesis of Kawasaki disease (44, 45). Stock et al. identified GM-CSF as an essential inflammatory cytokine in the development of cardiac inflammation during KD (44). When locally expressed GM-CSF switches on an inflammatory gene profile in resident macrophages of the heart, thus initiating cardiac disease. Another inflammatory pathway linked to PTPRJ was described by Wen et al. (46). It was found that PTPRJ-as1 significantly activated the NF-κB (Nuclear factor kappa-light-chain-enhancer of activated B cells) pathway in microglia under the influence of inflammatory environment and promoted the secretion of inflammatory cytokines: interleukin-6 (IL-6), tumor necrosis factor α (TNF-α) and inducible nitric oxide synthase (iNOS) and nitric oxide (NO) that was involved in inflammatory injury caused by intracerebral hemorrhage. The study by Tian et al. revealed that the expression of caspase-4, mediated by NF-κB signal pathway plays a critical role in KD (47). Human coronary artery endothelial cells (HCAEC) treated with supernatant conditioned by cells from KD patients showed a significant elevation of NF-κB p65 and caspase-4 protein expression vs. HCAEC cells treated with supernatant conditioned by control cells. We hypothesize that understanding the precise mechanism involved in the regulation of inflammation by PTRPJ in KD may be of significance.

### SPECC1L

The SPECC1L gene encodes a cytoskeletal crosslinking protein named sperm antigen with calponin homology and coiled-coil domains 1 like. It is involved in cytokinesis, spindle organization, actin cytoskeleton reorganization and microtubule stabilization and hence is required for proper cell adhesion and migration (48). Diseases associated with SPECC1L include congenital disorders: oblique facial clefting-1, Opitz GBBB syndrome type II, Teebi hypertelorism syndrome. The potential linkage between SPECC1L and the immune system or inflammation and Kawasaki disease is unknown.

### RPN2

This gene encodes ribophorin II (RPN2)—an integral membrane protein found only in the rough endoplasmic reticulum. This protein is part of an N-oligosaccharyltransferase complex that links high mannose oligosaccharides to asparagine residues found in the Asn-X-Ser/Thr consensus motif of nascent polypeptide chains. RPN2 is multifunctional; it has been demonstrated to be a prognostic marker of many cancers (49). RPN2 expression correlates with osteosarcoma, gastric adenocarcinoma and colorectal cancer (50–52). Takahashi et al. showed that RPN2 regulates tumor initiation and metastasis through the stabilization of mutant p53 in breast cancer cells (53). RPN2 is also highly expressed in the CD24+CD44+ cancer stem-like cells of pancreatic cancer (54). Huang L found that RPN2 promotes metastasis of hepatocellular carcinoma cell and inhibits autophagy via STAT3 (signal transducer and activator of transcription-3) and NF-κB pathways (55). The role of NF-κB in KD has been already mentioned. STAT3 is activated by interleukin 6, a pro-inflammatory cytokine that is involved in early innate immune reactivity, and present in the acute phase of KD (12, 56). Wang X et al. showed that microRNA (miR-223-3p) plays a protective role against endothelial injury in KD, by targeting IL6ST and by regulating the STAT3-NF-κB signaling pathway, making it a potential target for the diagnosis and treatment of KD (57). RPN2 is also involved in the binding of prothrombin to the monocyte surface. Fujieda suggested that RPN2 is involved in the pathophysiology of thrombosis in patients with antiphospholipid syndrome (APS) (58). Thrombosis in KD is well-known, but its relation with APS and antiphospholipid antibodies is still debated. It is worth to note that RPN2 could act via another inflammatory—JAK (Janus kinase) pathway. The Janus kinase/signal transducers and activators of transcription (JAK/STAT) axis is an inflammatory-associated pathway that is activated after receptor ligation, it mediates the signaling from cytokine receptors to the nucleus (59). Ni L et al. found that RPN2 targets Janus kinase 1 and promotes osteogenic differentiation of human bone mesenchymal stem cells (60). It is known that JAK1/STAT3 signaling pathway is activated in some systemic vasculitides (Behcet disease) through the activation of Th1/Th17-type cytokines such as IL-2, interferon (IFN-γ), IL-6, IL-17, and IL-23 but its role in KD is not well-understood (61). We speculate that the RPN2/JAK1/STAT3 axis could be a potential therapeutic target in Kawasaki disease because of its known effect on inflammatory reactions.

The most significant variant related to Kawasaki disease according to this study was rs12037447, which is placed in non-coding regulatory sequence and the analysis showed that this variant can potentially alter binding of 16 different regulatory motifs. One of the most interesting findings may be an altered P300 regulatory motif. P300 has been identified as a co-activator of HIF1α (hypoxia-inducible factor 1 alpha) in HIF-1 signaling pathway (GeneID: 2033). On the other hand, HIF-1 produced by macrophages and neutrophils is involved in the regulation of the inflammatory response and the intensification of the innate immune response. Moreover, HIF-1 signaling pathway leads to angiogenesis through activation of the VEGF signaling pathway. It has been previously reported that the serum level of vascular endothelial growth factor A (VEGF-A) in KD patients was correlated with the development of coronary arterial lesions (62). This suggests that this variant may play a role in disease development by influencing the regulatory function of P300. This polymorphism may also affect the enhancer binding protein beta (C/EBPβ). This is interesting because research suggests that C/EBPβ and C/EBPδ activation is associated with intravenous immunoglobulin therapy resistance in KD patients (63). Other altered regulatory motifs do not seem to have a connection to the pathogenesis of KD. Nevertheless, we believe that rs12037447 could be potentially connected with the pathogenesis of KD. Further studies are required to explain the complex effect of this variant.

Interestingly, we did not find the SNPs of known candidate genes for KD to be statistically significant in this analysis: rs361525 (TNF), rs1569723 (CD40), rs17531088 (NAALADL2), rs2078087 (NMNAT2) (Supplementary Table 1) (10, 11, 64, 65). The explanation for that might be the specificity of the GWAS method. Genome Wide Association Studies are a powerful tool to discover novel disease susceptibility genes, but it has its limitations. One of them is the requirement for a larger affected sample group; therefore, by increasing it, we could possibly observe SNPs of known genes in KD to be more statistically significant. Moreover, expanding the study group would allow us to perform more complex analysis, by differentiation of the study group into subgroups by adding secondary phenotypes, for example, information about a patient's resistance for intravenous immunoglobulin therapy and thus make an attempt to identify variants related to this phenomenon (66). Another secondary phenotype may be gender, as research suggests a link to male-specific association of polymorphisms in FCGR2A gene with Kawasaki disease (67). Other well-known SNPs related to the development of KD, such as rs1801274 (FCGR2A) (10), rs2254546 (FAM167A-BLK region) (8), rs2736340 (BLK) (64) were not included in the analysis, because they were filtered out at the beginning of workflow, due to multi-allelism which makes it impossible to predict a risk allele based solely on genotyping data. Despite this, the re-analysis was carried out containing these variants which showed that they did not occur statistically more or less frequently in this study group than in controls (unpublished data).

Following the obtained results, polymorphisms of genes KIF25, PTRPJ, SPECC1L, and RNP2 seemed to be involved in KD in Polish children. They also may explain the incidence of KD in Poland; however geographic distribution of gene polymorphisms in Polish children is unknown. This study's limitation is a relatively small sample size, thus multicenter studies on large sample sizes are needed to further reveal the relationship between the above mentioned genes and KD.

## Data Availability Statement

The original contributions generated in the study are included in the article/Supplementary Material, further inquiries can be directed to the corresponding authors.

## Ethics Statement

The studies involving human participants were reviewed and approved by The Bioethics Committee at the Children's Memorial Health Institute in Warsaw. Written informed consent to participate in this study was provided by the participants' legal guardian/next of kin.

## Author Contributions

PB: conceptualization, methodology, validation, investigation, formal analysis, writing—original draft preparation, visualization, supervision, and project administration. MC: methodology, validation, formal analysis, resources, software, data curation, writing—original draft preparation, visualization, and supervision. AS-K, AW-K, AP, MK-D, and MO-N: investigation, writing—review, and editing. MS-K: methodology and data curation. ŁG: methodology, writing—review, and editing. MS and AS: data curation. JK: writing—review and editing. DS: methodology, validation, formal analysis, resources, funding acquisition, visualization, supervision, writing—review, and editing. All authors have read and agreed to the published version of the manuscript.

## Conflict of Interest

The authors declare that the research was conducted in the absence of any commercial or financial relationships that could be construed as a potential conflict of interest.
