# Supplementary material for: Association Between rs12037447, rs146732504, rs151078858, rs55723436, and rs6094136 Polymorphisms and Kawasaki Disease in the Population of Polish Children
Source: Front Pediatr. 2021 Feb 22;9:624798. doi: 10.3389/fped.2021.624798 (PMC7937642; doi:10.3389/fped.2021.624798)
Supplement: Supplementary file 1 [file Data_Sheet_1.docx]

**Supplementary Information**

Association between rs12037447, rs146732504, rs151078858, rs55723436 and rs6094136 polymorphisms and Kawasaki disease in the population of Polish children

***Piotr Buda*^1#^, Maciej Chyb^2#^, Anna Smorczewska-Kiljan^1^, Anna Wieteska-Klimczak^1^, Agata Paczesna^3^, Monika Kowalczyk-Domagała^3^, Magdalena Okarska-Napierała^4^, Marta Sobalska-Kwapis^2,5^, Łukasz Grochowalski^2^ ,Marcin Słomka^2,5^,*** ***Aneta Sitek^6^ , Janusz Książyk^1^, Dominik Strapagiel*^2,5^***

1. Department of Pediatrics, Nutrition and Metabolic Diseases, Children’s Memorial Health Institute, Warsaw, Poland

2. Biobank Lab, Department of Molecular Biophysics, Faculty of Biology and Environmental Protection, University of Lodz, Lodz, Poland

3. Department of Cardiology, The Children’s Memorial Health Institute, Warsaw, Poland

4. Department of Pediatrics with Clinical Decisions Unit, Medical University of Warsaw, Warsaw, Poland

5. BBMRI.pl Consortium, Wroclaw, Poland

6. Department of Anthropology, Faculty of Biology and Environmental Protection, University of Lodz, Lodz, Poland.

**# Contributed equally**

*** Correspondence:**

Piotr Buda:Department of Pediatrics, Nutrition and Metabolic Diseases, Children’s Memorial Health Institute, 04-730 al. Dzieci Polskich 20, Warsaw, Poland; e-mail: [p.buda@ipczd.pl](mailto:p.buda@ipczd.pl)

Dominik Strapagiel: Biobank Lab, Department of Molecular Biophysics, Faculty of Biology and Environmental Protection, University of Łódź, 90-231 ul. Pilarskiego 14/16 Łódź, Poland; e-mail: [dominik.strapagiel@biol.uni.lodz.pl](mailto:dominik.strapagiel@biol.uni.lodz.pl)

**Suplementary Table 2**. SIFT prediction results

| SNP | CHR/POS | REF/ALT ALLELE | AMINO ACID CHANGE | GENE NAME | GENE_ID | TRANSCRIPT ID | PROTEIN ID | REGION | SIFT SCORE | SIFT MEDIAN | NO OF SEQS AT POSITION | SIFT PREDICTION |
| --- | --- | --- | --- | --- | --- | --- | --- | --- | --- | --- | --- | --- |
| rs146732504 | 6:168434704 | C/A | L9I | KIF25 | ENSG00000125337 | ENST00000496008 | ENSP00000427464 | CDS |  |  |  |  |
| rs146732504 | 6:168434704 | C/A | L104I | KIF25 | ENSG00000125337 | ENST00000354419 | ENSP00000346401 | CDS | 0.012 | 2.62 | 39 | DELETERIOUS |
| rs146732504 | 6:168434704 | C/A | L104I | KIF25 | ENSG00000125337 | ENST00000443060 | ENSP00000388878 | CDS | 0.012 | 2.62 | 39 | DELETERIOUS |
| rs146732504 | 6:168434704 | C/A | L104I | KIF25 | ENSG00000125337 | ENST00000351261 | ENSP00000252688 | CDS | 0.094 | 2.5 | 33 | TOLERATED |
| rs151078858 | 11:48177395 | C/T | T1091M | PTPRJ | ENSG00000149177 | ENST00000418331 | ENSP00000400010 | CDS | 0.035 | 2.52 | 37 | DELETERIOUS |
| rs55723436 | 22:24718408 | G/A | R487H | SPECC1L | ENSG00000100014 | ENST00000541492 | ENSP00000439633 | CDS | 0.019 | 2.61 | 26 | DELETERIOUS |
| rs55723436 | 22:24718408 | G/A | R487H | SPECC1L | ENSG00000100014 | ENST00000314328 | ENSP00000325785 | CDS | 0.064 | 2.56 | 26 | TOLERATED |
| rs55723436 | 22:24718408 | G/A | R487H | SPECC1L | ENSG00000100014 | ENST00000437398 | ENSP00000393363 | CDS | 0.064 | 2.56 | 26 | TOLERATED |
| rs12037447 | Not | found |  |  |  |  |  |  |  |  |  |  |
| rs6094136 | Not | found |  |  |  |  |  |  |  |  |  |  |

**Supmenetary Table 3.** Regulatory motifs altered for rs12037447 (HaploReg v4.1) (Pouya Kheradpour, Manolis Kellis, Systematic discovery and characterization of regulatory motifs in ENCODE TF binding experiments, Nucleic Acids Research, Volume 42, Issue 5, 1 March 2014, Pages 2976–2987)

| Position Weight Matrix ID (Library from Kheradpour and Kellis, 2013) | Strand | Ref | Alt | Match on:  Ref: CAAAATGCCTGAGTTTGGAGATAAATTGCATAATTGTTCTATGCATAGGCATGGGAAAT Alt: CAAAATGCCTGAGTTTGGAGATAAATTGCGTAATTGTTCTATGCATAGGCATGGGAAAT |
| --- | --- | --- | --- | --- |
| AFP1 | + | 13.7 | 1.8 | AWWAAYWRSAY |
| CEBPA_1 | + | 10.7 | 10 | HNRTTRSNHMANHH |
| CEBPA_2 | + | 11.2 | 12 | RTTGYRHMW |
| CEBPB_disc1 | + | 12.9 | 13.9 | RTTGYRCAAY |
| CEBPB_known1 | - | 11.1 | 10.6 | DDVTKDDGHAAN |
| CEBPB_known2 | + | 11.2 | 10.5 | HRKVBMTTRCVDMABWCN |
| CEBPD | + | 14.7 | 15.5 | VATTDCDYMMYY |
| Dlx2 | - | 11.5 | 12 | NTDDDVYAATTRYHBB |
| En-1_1 | + | 7.4 | 8.6 | RWDDTBB |
| HLF | - | 12.1 | 14.3 | ATKRYGTAAY |
| Hoxa3_1 | + | 8 | 8.7 | SVTWWDDKB |
| Nkx2_8 | - | 13.3 | 2.6 | SWTAATWV |
| Pou1f1_2 | - | 12.5 | 1 | HMTKCWTDHWBRWHHHHH |
| Pou2f2_known1 | - | 7.5 | -4.5 | MKVATTWGCATAYY |
| Pou2f2_known10 | - | 11.3 | 6.5 | VKDMTTTRCATDBHB |
| Pou2f2_known7 | - | 12.6 | 0.7 | DNATTTRCATD |
| Pou3f3 | - | 11.9 | 5.4 | HWDDTATGCATAWDDDW |
| STAT_disc4 | - | 4.7 | 3.7 | TTGWGYAAT |
| Sox_16 | - | 12.2 | 12 | DNDDYMATTGTYVHDH |
| Sox_19 | - | 12 | 11.4 | NDWWBWATTGTTHHDWW |
| Sox_2 | - | 12.6 | 12.4 | DNDWNNDYHATTGTTHHDHVDD |
| Sox_9 | - | 12.2 | 11.2 | HDHHWATTGTTCHHDH |
| TATA_disc9 | + | 11.4 | 6.3 | ATWTGCAWAW |
| p300_disc2 | + | 11.8 | 6.4 | NRTTKCAHMABHHHH |
